# Light Intensity Alters the Behavior of *Monilinia* spp. *in vitro* and the Disease Development on Stone Fruit-Pathogen Interaction

**DOI:** 10.3389/fpls.2021.666985

**Published:** 2021-09-08

**Authors:** Marta Balsells-Llauradó, Rosario Torres, Núria Vall-llaura, Carla Casals, Neus Teixidó, Josep Usall

**Affiliations:** IRTA, Postharvest Programme, Edifici Fruitcentre, Parc Científic i Tecnològic Agroalimentari de Lleida, Parc de Gardeny, Lleida, Spain

**Keywords:** necrotroph, brown rot, nectarine, photomorphogenesis, preharvest, postharvest, bagging, ethylene

## Abstract

The development of brown rot caused by the necrotrophic fungi *Monilinia* spp. in stone fruit under field and postharvest conditions depends, among others, on environmental factors. The effect of temperature and humidity are well studied but there is little information on the role of light in disease development. Herein, we studied the effect of two lighting treatments and a control condition (darkness) on: (i) several growth parameters of two *Monilinia* spp. (*M. laxa* and *M. fructicola*) grown *in vitro* and (ii) the light effect in their capacity to rot the fruit (nectarines) when exposed to the different lighting treatments. We also assessed the effect of such abiotic factors in the development of the disease on inoculated nectarines during postharvest storage. Evaluations also included testing the effect of fruit bagging on disease development as well as on ethylene production. Under *in vitro* conditions, lighting treatments altered colony morphology and conidiation of *M. laxa* but this effect was less acute in *M. fructicola*. Such light-induced changes under *in vitro* development also altered the capacity of *M. laxa* and *M. fructicola* to infect nectarines, with *M. laxa* becoming less virulent. The performance of *Monilinia* spp. exposed to treatments was also determined *in vivo* by inoculating four bagged or unbagged nectarine cultivars, indicating an impaired disease progression. Incidence and lesion diameter of fruit exposed to the different lighting treatments during postharvest showed that the effect of the light was intrinsic to the nectarine cultivar but also *Monilinia* spp. dependent. While lighting treatments reduced *M. laxa* incidence, they enhanced *M. fructicola* development. Preharvest conditions such as fruit bagging also impaired the ethylene production of inoculated fruit, which was mainly altered by *M. laxa* and *M. fructicola*, while the bag and light effects were meaningless. Thus, we provide several indications of how lighting treatments significantly alter *Monilinia* spp. behavior both *in vitro* and during the interaction with stone fruit. This study highlights the importance of modulating the lighting environment as a potential strategy to minimize brown rot development on stone fruit and to extent the shelf-life period of fruit in postharvest, market, and consumer’s house.

## Introduction

Species of *Monilinia* are responsible of brown rot disease on stone fruit both in the field and during postharvest. In particular, *Monilinia laxa* is found worldwide (Obi et al., 2018) and is the main causal agent of brown rot in Europe (Rungjindamai et al., 2014), while *Monilinia fructicola* is more virulent (Kreidl et al., 2015) and its presence has been increasing in Spanish orchards since 2006 (De Cal et al., 2009; Villarino et al., 2013). These pathogens are necrotrophic since they can colonize fruit tissues causing cellular death (Garcia-Benitez et al., 2016), rotting most parts of the tree, from buds to fruit (Villarino et al., 2010). Sources of inoculum can be primary [e.g., from mummified fruit (Gell et al., 2008)] or secondary [e.g., from infected fruit (Villarino et al., 2012)], resulting in a polycyclic disease (reviewed in Oliveira Lino et al., 2016).

Environmental conditions are critical for brown rot development. Temperature and wetness period are the most studied factors and are demonstrated to influence penetration and spread of both *M. laxa* (Gell et al., 2008) and *M. fructicola* (Luo and Michailides, 2001). Solar radiation, wind speed and rainfall factors also play an important role in the spread of *M. laxa* and *M. fructicola* (Gell et al., 2009) but detailed information is scarce. During plant-pathogen interactions, light quantity and quality (Idnurm and Crosson, 2009) and photoperiod (Tisch and Schmoll, 2010) not only influence the behavior of the pathogen, but also the interaction with its hosts (Carvalho and Castillo, 2018).

Fungi are able to adapt their metabolic pathways when perceiving light (Tisch and Schmoll, 2010; Corrochano, 2019) through a complex of photoreceptors and so regulate their behavior and development (Bahn et al., 2007), such as the development of sexual or vegetative reproductive structures and tropism of unicellular structure (Corrochano, 2019). The light alters gene expression patterns of *Monilinia* spp. (De Miccolis Angelini et al., 2018) and, in fact, some photoreceptors and related regulatory proteins (e.g., velvet regulatory family) have recently been described in *M. laxa* (Rodríguez-Pires et al., 2021). However, how the fungus perceives and modulates light responses needs further investigation. For instance, *Botrytis cinerea*, a species of the same family of *Monilinia* spp., produces sclerotia in constant darkness and conidia under the light, which is enhanced when growing under light-dark cycles compared to constant light (Schumacher, 2017). Hence, the presence of light but also its intensity, quality and photoperiod can alter fungal development both under *in vitro* conditions and on fruit. Thus said, little is known regarding how light can affect the infection process of phytopathogenic fungi, and only one study incubating *M. laxa*-inoculated stone fruit under different white light conditions and photoperiods have been conducted (Rodríguez-Pires et al., 2020).

Likewise, light regulates plant growth and development (Folta and Carvalho, 2015), including responses to biotic stresses (Roeber et al., 2020). Perception of light can control the establishment of the systemic acquired resistance, which would lead to an enhancement of disease resistance in several plant-pathogen interactions (Métraux, 2002; Roberts and Paul, 2006). When both the pathogen-host interaction and light conditions take place, plant circadian rhythm controls the pathogen host, leading to a daytime-dependent response (Griebel and Zeier, 2008). After an interaction, the host induces a hormone signaling cascade, which in turn, triggers defense mechanisms (Pandey et al., 2016). Ethylene is one of the multiple hormones which mediates the host response against necrotrophic pathogens (McDowell and Dangl, 2000), although it also modulates the response to numerous abiotic stresses (reviewed in Müller and Munné-Bosch, 2015). In fact, recent studies have demonstrated the link between the jasmonate/ethylene pathway and the photoreceptor-mediated light response, and its importance on the resistance to the pathogen *B. cinerea* (Xiang et al., 2020).

The solar radiation that fruit receives in the field varies along the year, being low at the beginning and higher at the end of the stone fruit season. Nowadays, growers are implementing some alternative practices to control pests and diseases during preharvest (Usall et al., 2015) in substitution to those based on chemicals. Among them, fruit bagging (Allran, 2017) has been proved to be effective in controlling brown rot incidence in peach and plum (Keske et al., 2011, 2014). However, these alternatives, together with the use of colored shade nets (Ilić and Fallik, 2017), have also an effect on the incidence of solar radiation that fruit receives during its development, altering many fruit physicochemical properties (Sharma et al., 2014; Ilić and Fallik, 2017; Zhou et al., 2019), which ultimately could impair the fruit response to pathogens.

Therefore, the understanding of the light effect on the pathogens but also on the capacity of fruit to respond properly to infections is critical to establish an optimal practice in the field but also along the postharvest in packinghouses and through the distribution chain. Thus said, this study aimed to understand the effect of the darkness (control) and two lighting treatments on the behavior of *M. laxa* and *M. fructicola in vitro* and during the interaction with nectarine fruit. In particular, we assessed the (*i*) effect of the three treatments on the ecophysiology of *Monilinia* spp. *in vitro* in two different culture media; (*ii*) effect of the three treatments in the capacity of the two species after being exposed to the lighting treatments to infect fruit; (*iii*) effect of fruit bagging on fruit susceptibility to brown rot at postharvest; (*iv*) effect of the three treatments in the ethylene production and the development of the disease of the inoculated fruit exposed to different lighting treatments.

## Materials and Methods

### Fungal Material and Incubation Treatments

The species of *Monilinia* used in this study were single-conidia strains of *M. laxa* (ML8L) and *M. fructicola* (CPMC6), deposited in the Spanish Culture Type Collection (CECT 21100 and CECT 21105, respectively). Fungal cultures and conidial suspensions were maintained and prepared as described by Baró-Montel et al. (2019c). Fungal suspensions were prepared at 10^5^ conidia mL^–1^ and used to inoculate plates or fruit depending on the experiment.

Both *in vitro* and *in vivo* experiments were conducted in a growth chamber with the following incubation and lighting treatments: (1) “Control”, at 20°C, 45–55 % RH and complete darkness; (2) “Treatment 1,” consisting of 4 fluorescents of low light intensity and incandescent white TL-D 36 W/827 (Ta = 2700 K, 3350 lm, 350 – 740 nm, 630 nm max) (Philips), and photoperiod of 12 h light (22 ± 1°C, 50 ± 10 % RH) / 12 h dark (20°C, 90 % RH); (3) “Treatment 2,” consisting of 4 fluorescents of high light intensity and cool white TL-D 58W/840 (Ta = 4000 K, 5000 lm, 300 – 740 nm, 550 nm max) (Philips), and photoperiod of 16 h light (21 ± 1°C, 50 ± 10 % RH) / 8 h dark (20°C, 90 % RH).

### *In vitro* Ecophysiology

To evaluate the light effect on the two strains of *Monilinia* spp., Potato Dextrose Agar (PDA; Biokar Diagnostics, 39 g L^–1^) and/or PDA plates supplemented with 25 % tomato pulp (PDA-T) were inoculated with one drop of 10 μL of the conidial suspension (10^5^ conidia mL^–1^) of each species on the center of Petri dishes. Plates were incubated under the three incubations treatments mentioned above. During and after 7 days under each treatment, ecophysiological parameters for both species were evaluated: growth parameters (including colony morphology, conidiation, conidia morphology, and growth rate), conidial viability and germination. All experiments consisted of three replicates per treatment, culture media and *Monilinia* spp. and each experiment was repeated twice.

#### Growth Parameters

Four growth parameters were investigated for each *Monilinia* spp. grown in PDA and PDA-T media. The colony growth rate, the total conidiation, a visual inspection of colony features according to EPPO standard PM 7/18 (3) (Bulletin OEPP/EPPO, 2020) and the conidia morphology of cultures were assessed. The colony growth rate (cm day^–1^) was determined as the slope of the lineal equation obtained from the individual measurements of the mean of the colony diameter in two perpendicular directions by plotting growth diameter (cm) *vs.* time (days). Conidiation was calculated by rubbing the conidia from the surface of the PDA-T plates with a known volume of sterile water containing 0.01 % Tween-80 (*w*/*v*), filtering through two layers of sterile cheesecloth and then titrating the conidia using a haemocytometer. The concentration of conidiation (conidia mL^–1^) was calculated and expressed as total conidiation in relation to control. Comparison of conidia morphology from plates subjected to different treatments was assessed by rubbing the PDA and PDA-T plates with sterile water containing 0.01 % Tween-80 (*w*/*v*) and filtering through two layers of sterile cheesecloth. Images at 40x magnification were taken in an optical microscope (Leica DM5000B, Leica Microsystems CMS GmbH, Germany). The images were acquired using a Leica color digital camera (Leica DFC 420).

#### Conidial Viability

To test the conidial viability (i.e., the ability of conidia to form new colonies) after exposing the *Monilinia* spp. grown in PDA-T media for 7 days under the different light regimes, colony-forming units (CFUs) were measured by performing serial ten-fold dilutions on PDA medium. Plates were incubated for 3 to 4 days at 20°C under darkness.

#### Germination of Conidia

Percentage of germinated conidia (%) was studied under optical microscopy, as described by Casals et al. (2010) with some modifications. Droplets (10 μL) of the conidial suspension (10^5^ conidia mL^–1^) were placed around PDA plates, and immediately incubated under each treatment. Samplings were carried out each 30 min or 1 h until 6 h. To stop germination at each incubation time, 1 mL of 25 % ammonia was applied onto a filter paper placed on the cover of the Petri dish. Conidia were considered germinated when cell wall deformation forming a germ tube was observed.

### Light Effect on the Ability of *Monilinia* spp. to Infect Fruit

To evaluate whether the capacity of *Monilinia* spp. to infect fruit was altered by treatments, an inoculation of nectarines with the two species previously exposed to the three treatments was conducted. Experiments were performed with two organically grown cultivars of nectarines [*P. persica* var. *nucipersica* (Borkh.) Schneider]. “Fantasia” and “Venus” cultivars were obtained from an orchard located in Alfarràs and Ivars de Noguera (Lleida, Catalonia, Spain), respectively. Fruit for analysis was further homogenized by using a portable DA-Meter (TR-Turoni, Forli, Italy), based on the single index of absorbance difference.

#### Fruit Inoculations

Cultures of *Monilinia* spp. exposed to each treatment were used to artificially inoculate nectarines. One drop (10 μL) of conidial suspensions (10^5^ conidia mL^–1^) of *M. laxa* or *M. fructicola* was placed on PDA-T plates and cultures were maintained under each afore-mentioned treatment (section “Fungal material and incubation treatments”) for 7 days. Conidial suspensions of both species were prepared as described above (section “Fungal material and incubation treatments”). Non-wounded fruit was inoculated with one drop (10 μL) of conidial suspension (10^5^ conidia mL^–1^). A total of 20 fruits per cultivar, species, and treatment were used. Fruit were stored in a growth chamber, inside plastic boxes with wet filter paper (distilled water), under darkness and controlled incubation conditions (20 ± 1°C, 97 ± 3 % RH).

#### Aggressiveness Parameters

Disease symptoms were examined to calculate incidence (percentage of fruit with brown rot symptoms) and severity (lesion diameter length in cm of rotted fruit) along 7 days after inoculation. The incubation period (number of days to the observation of the onset of brown rot symptoms) and the latency period (number of days to the observation of conidiation) were also recorded. In fruit inoculated with *M. fructicola*, the conidiation was determined on the fruit surface after 7 days post-inoculation (dpi) for each treatment. For that, peels of the infected area of 3–4 inoculated fruits were obtained, immersed in a sterile filter bag with 40 mL sterile water containing 0.01 % Tween-80 (*w*/*v*) and homogenized in a Stomacher (Seward, London, United Kingdom) set at 12 strokes s^–1^ for 120 s. The filtered volume was recovered and the conidia was counted using a haemocytometer. The concentration of conidia (conidia g fresh peel^–1^) was calculated as the mean of each group of 3–4 fruit.

### Light Effect on the *Monilinia* spp.-Fruit Interaction

To evaluate the light effect on the interaction of *Monilinia* spp. with nectarine, inoculated fruit with both *M. laxa* and *M. fructicola* were incubated under each afore-mentioned treatment. For that, experiments were conducted with organically grown cultivars of nectarines; two early-mid (“Fantasia” and “Venus”) and two late (“Nectatinto” and “Albared”) cultivars, obtained from an orchard located in Alfarràs, Ivars de Noguera, Gimenells and Alfarràs (Lleida, Catalonia, Spain), respectively. The light effect was assessed on unbagged and bagged fruit, which was bagged with white paper bags at least one month before harvest. Bagged and unbagged fruit were harvested in the same sun-side of trees due to the influence of fruit canopy position to all fruit’s characteristics (Minas et al., 2018). Bags were removed just before conducting assays. Fruit for analysis was further homogenized by using a portable DA-Meter (TR-Turoni, Forli, Italy), based on the single index of absorbance difference.

#### Fruit Inoculations and Conidia Establishment

Inoculation was carried out by placing one drop (50 μL) of the conidial suspension (10^5^ conidia mL^–1^) on the colored side of non-wounded fruit. A mock inoculation (mock) was performed by inoculating sterile water containing 0.01 % Tween-80 (*w*/*v*). Inoculated fruit was first incubated at high humidity conditions for 24 h for the establishment of conidia on the fruit surface. For that, fruit was placed on boxes covered with a wet paper and a plastic bag, and then stored in a growth chamber, at controlled conditions (20°C, 90 ± 3 % RH). After that, fruit were immediately placed under each lighting treatment.

#### Aggressiveness Parameters and Ethylene Measurements

Fruit were daily examined to calculate brown rot incidence, severity and incubation period along 7 days, as described above (section “Aggressiveness parameters”). Experiments were conducted with 4 replicates of 5 fruits each per cultivar, bagging condition, treatment and *Monilinia* spp. Ethylene production of both mock-inoculated fruit and *Monilinia* spp. inoculated fruit was determined as described by Giné-Bordonaba et al. (2017). Measurements were conducted at four time points along the infection time course until 7 dpi. At each sampling point, fruit were placed in 3.8 L sealed flasks and left to incubate for 2 h. After ethylene measurements, fruit were placed back under each lighting treatment. Experiments were conducted with four replicates of three fruits each.

### Statistical Analysis

Data were statistically analyzed with JMP software version 14.2.0 (SAS Institute Inc., Cary, NC, United States). Prior to the analysis, all data were checked for the assumptions of parametric statistics and transformed when needed. Data of *in vitro* assays (growth rate, total conidiation and conidial viability), conidiation on fruit surface and severity were used as original data. Incubation and latency period (dpi) were subjected to square root transformation. Data of ethylene production (μL kg^–1^ h^–1^) were subjected to Log transformation. All these data were subjected to analysis of variance (ANOVA). Conidia germination (%) was analyzed using the generalized linear model (GLM) based on a Poisson distribution and Log-link function. Brown rot incidence (%) was analyzed using the GLM based on a binomial distribution and logit-link function. When the analysis was statistically significant, orthogonal contrasts (*P* ≤ 0.05) were performed for means separation among treatments. When comparisons were conducted between two means (bagged *vs.* unbagged), Student’s *T*-test (*P* ≤ 0.05) was used. For means comparison of inoculated fruit (mock, *M. laxa* and *M. fructicola*), Tukey’s HSD test (*P* ≤ 0.05) was conducted.

## Results

### Light Differentially Alters the Phenotype of *M. laxa* and *M. fructicola*

**FIGURE 1:**
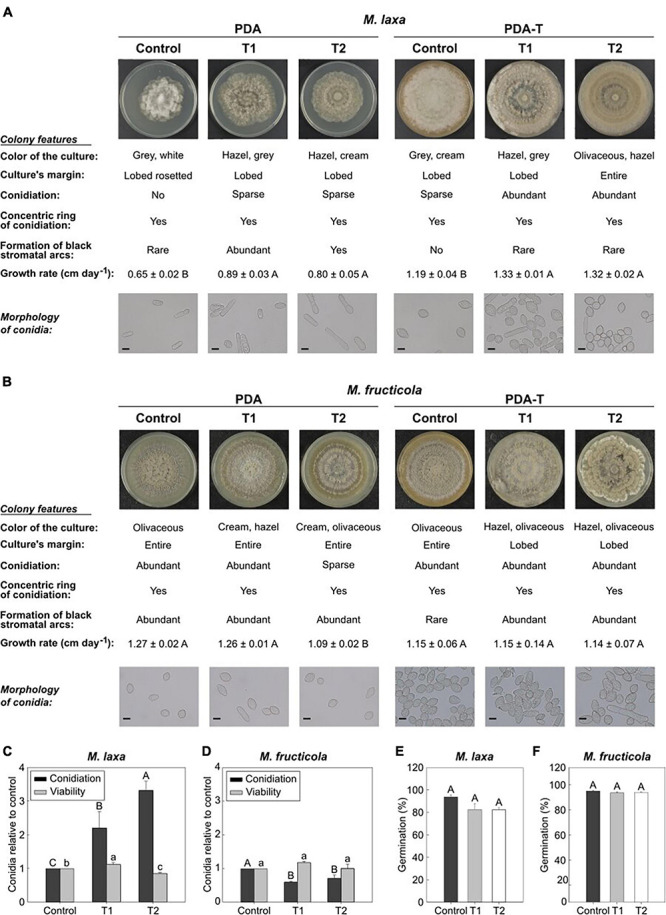
*In vitro* ecophysiology of *Monilinia* spp. after exposure to treatments 1 and 2 and control condition (constant darkness). Images of *Monilinia* cultures, description of colony features, growth rate (cm day^–1^) and microscopy images (40×) of *M. laxa* **(A)** and *M. fructicola* **(B)** grown on PDA and PDA-T and incubated under each light condition. Data for growth rate represent the mean of replicates (*n* = at least 4) ± standard error of the means. Different letters indicate significant differences (*P* ≤ 0.05) among incubation conditions according to orthogonal contrasts. Scale bar for microscopy images is indicated (10 μm). Conidiation and conidial viability of *M. laxa* **(C)** and *M. fructicola* **(D)** grown on PDA-T incubated under each light condition. Data is represented relative to the control condition (control = 1). Different uppercase and lowercase letters indicate significant differences (*P* ≤ 0.05) of conidiation and conidial viability, respectively, among incubation conditions according to orthogonal contrasts. Germination (%) after 6 h of *M. laxa* **(E)** and *M. fructicola* **(F)** on PDA medium. Different letters indicate significant differences (*P* ≤ 0.05) among incubation conditions according to orthogonal contrasts. For panels **(C–F)**, bars represent the mean of replicates (*n* = at least 4) and error bars represent the standard error of the means.

To evaluate the light effect on the *in vitro* behavior of *Monilinia* spp., we assessed several ecophysiological parameters after exposing *M. laxa* and *M. fructicola* to two lighting treatments and control condition (constant darkness) for 7 days (Figure 1). Under both treatments, colony features were very different from those grown under control condition, for each *Monilinia* spp. in both culture media (Figures 1A,B). The colonies of *M. laxa* in both culture media subjected to both lighting treatments showed more hazel colors if compared to those white and gray colors observed in the control condition. *Monilinia laxa* significantly grew faster under both lights than under control condition in both media. *Monilinia fructicola* grown on PDA-T and subjected to both lighting treatments presented lobed culture’s margin, while when growing under control condition, colonies presented entire margins. Only treatment 2 was able to significantly reduce its growth rate when growing on PDA but not in PDA-T medium. Conidia morphology examination showed that, except for *M. laxa* on PDA where few conidia were detected, both treatments altered conidia shapes of both *M. laxa* and *M. fructicola* (Figures 1A,B). While conidia from control condition cultures presented the typical ovoid and limoniform morphologies, lighting treatments induced an increase of irregular morphologies such as globose, cylindrical, or ellipsoidal (Figures 1A,B).

The visual inspection of *Monilinia* cultures demonstrated that *M. laxa* produced more conidia in PDA-T plates exposed to both treatments 1 and 2 (2.19 and 3.31-fold significantly higher, respectively) if compared to control condition (constant darkness) (Figure 1C). However, we were not able to observe *M. laxa* conidiation on the PDA medium incubated under control condition (Figure 1A). In fact, almost no conidia were visualized in microscopic inspections in PDA plates (Figure 1A) as exposed above. In contrast, conidiation of *M. fructicola* was significantly reduced in PDA-T plates exposed to both treatments 1 and 2 (0.59 and 0.71-fold, respectively) if compared to control condition (Figure 1D). Conidiation in PDA plates was like that on PDA-T plates, where both treatments 1 and 2 significantly reduced (0.43 and 0.29-fold, respectively) the number of conidia in illuminated plates compared to control condition. Regarding the conidial viability, results showed that treatment 2 significantly reduced the number of CFUs of *M. laxa*, although on treatment 1 it was slightly higher (1.12-fold) than on control condition (Figure 1C). In contrast, we did not observe any effect of lighting treatment on the conidial viability of *M. fructicola* (Figure 1D). Finally, exposition to light affected the germination’s capability of neither *M. laxa* (Figure 1E) nor *M. fructicola* (Figure 1F).

### Contrary to *M. fructicola*, *M. laxa* Becomes Less Virulent Once Exposed to Lighting Treatment

**FIGURE 2:**
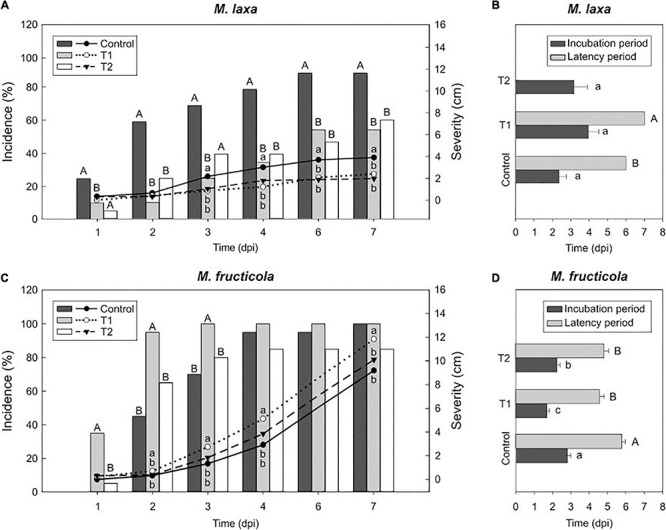
Light effect on the capacity of *Monilinia* spp. to infect fruit in “Fantasia” cultivar. Incidence (% of brown rot, bars) and severity (lesion diameter length in cm of rotted fruit, lines) of *M. laxa* **(A)** and *M. fructicola* **(C)** in “Fantasia” nectarines along the infection time course (dpi, days post-inoculation) after growing the fungi for 7 days under treatments 1 and 2 and control condition (constant darkness). Bars represent the mean of incidence on fruit (*n* = 20). Lines represent the mean of diameter length of rotted fruit. Different uppercase and lowercase letters indicate significant differences (*P* ≤ 0.05) of incidence and severity, respectively, among incubation conditions according to orthogonal contrasts at each time point. No letters indicate no significant differences. Incubation and latency periods (dpi) of *M. laxa* **(B)** and *M. fructicola* **(D)** in “Fantasia” nectarines after growing the fungi for 7 days under treatments 1 and 2 and control condition. Bars represent the mean of fruits with symptoms (*n* = 2 to 20) and error bars represent the standard error of the means. Different lowercase and uppercase letters indicate significant differences (*P* ≤ 0.05) of incubation and latency periods, respectively, among incubation conditions according to orthogonal contrasts.

To test how changes observed under *in vitro* ecophysiological parameters affected the capacity of both *Monilinia* spp. to infect fruit, we assessed the development of the disease on nectarines inoculated with *M. laxa* or *M. fructicola* which were previously grown under each lighting treatment. In ‘Fantasia’ nectarines inoculated with *M. laxa*, both treatments 1 and 2 significantly reduced incidence (55 and 61 %, respectively) and severity (2.4 and 2.0 cm, respectively) since the first time point compared to control condition (constant darkness) (90 % of incidence and 3.9 cm of severity) (Figure 2A). No differences in the incubation period were observed among treatments (Figure 2B). Only 10% of fruit inoculated with *M. laxa* which was grown under treatment 1 and control condition showed conidiation on the fruit surface. In line with these results, fruit inoculated with *M. laxa* grown under treatment 1 revealed a higher latency period (1.17-fold) than those inoculated with the pathogen held under control condition (Figure 2B). Besides, under the treatment 2, the fungal development did not even show any conidiation (Figure 2B). Thus, although both treatments improved the behavior of *M. laxa in vitro*, they made the pathogen impair and delay its capacity to infect and in consequence, made it less virulent.

Regarding *M. fructicola*, the incidence of fruit inoculated with *M. fructicola* grown under treatment 1 significantly peaked at early time points (up to 100 %), although such differences completely subsided through time (Figure 2C). Interestingly, only *M. fructicola* subjected to that treatment 1 was able to cause significantly higher lesion diameter on fruit (up to 11.8 cm) than that in the two other conditions (9.2 cm under control condition and 10.1 cm under treatment 2) (Figure 2C). In addition, both treatments accelerated the onset of disease symptoms. The incubation periods of fruit inoculated with *M. fructicola* exposed to treatment 2 and treatment 1 were significantly lower (1.25 and 1.65-fold, respectively) than when the pathogen was grown under control condition (constant darkness) (Figure 2D). Between 94 and 100% of inoculated fruit, irrespective of treatment in which the fungus was grown, presented conidiation on the fruit surface. However, the latency of *M. fructicola* under either lighting treatment significantly accelerated the onset of conidiation symptoms (between 4.6 and 4.8 days of average) compared to control condition (an average of 5.8 days) (Figure 2D). Finally, regarding the concentration of conidia in the fruit surface, treatment 1 induced *M. fructicola* to produce significantly more conidia on fruit (1.92-fold) compared to control condition, whereas treatment 2 was like control condition (Supplementary Figure 1). Hence, while lighting treatments seemed to make *M. laxa* lose virulence, it accelerated the onset of disease symptoms and conidiation of *M. fructicola.* All these experiments were also conducted in another nectarine cultivar (“Venus”) and results showed similar tendencies of fruit susceptibility to brown rot (Supplementary Figure 2).

### Fruit Bagging Can Alter Its Susceptibility to *Monilinia* spp. in a Cultivar-Dependent Manner

To test the effect of fruit bagging on fruit susceptibility to brown rot, we conducted a disease evaluation of four different nectarine cultivars inoculated with *M. laxa* and *M. fructicola* and incubated under control condition (constant darkness). In inoculated fruit with either *M. laxa* or *M. fructicola*, results showed two tendencies of fruit susceptibility (Supplementary Table 1). Unbagged “Fantasia” nectarines were more susceptible to both *Monilinia* spp. than fruit that was bagged during preharvest (“bagged fruit”). However, the other cultivars (“Venus,” “Nectatinto” and “Albared”) showed that unbagged fruit was slightly more resistant to both *Monilinia* spp. than bagged fruit. Hence, results pointed out that the effect of fruit bagging in fruit susceptibility to brown rot could be cultivar-dependent.

### Light Reduces *M. laxa* Disease in Nectarines but Enhance *M. fructicola* Development

**FIGURE 3:**
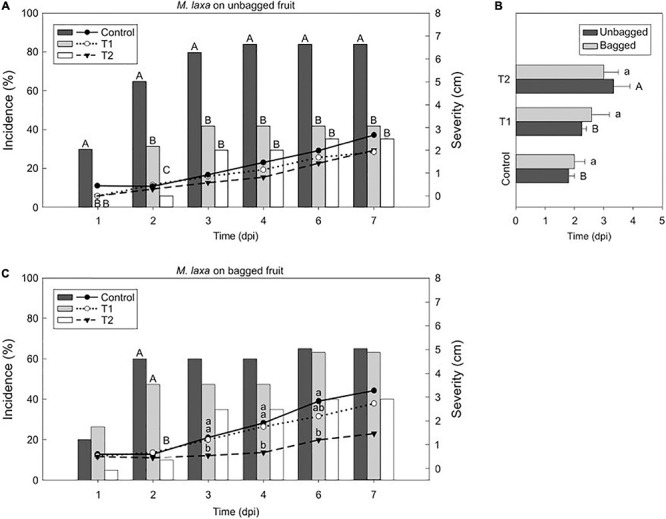
Light effect on the *M. laxa*-nectarine interaction. Incidence (% of brown rot, bars) and severity (lesion diameter length in cm of rotted fruit, lines) of *M. laxa* in unbagged **(A)** and bagged **(C)** “Fantasia” nectarines along the infection time course (dpi, days post-inoculation) incubated for 7 days under treatments 1 and 2 and control condition (constant darkness). Bars represent the mean of incidence on fruits (*n* = 20). Lines represent the mean of diameter length of rotted fruit. Different uppercase and lowercase letters indicate significant differences (*P* ≤ 0.05) of incidence and severity, respectively, among incubation conditions according to orthogonal contrasts at each time point. No letters indicate no significant differences. The incubation period (dpi) of *M. laxa* in bagged and unbagged “Fantasia” nectarines **(B)** after 7 days of incubation under treatments 1 and 2 and control condition. Bars represent the mean of fruits with symptoms (*n* = 2 to 20) and error bars represent the standard error of the means. Different uppercase and lowercase letters indicate significant differences (*P* ≤ 0.05) among incubation conditions in unbagged and bagged fruit, respectively, according to orthogonal contrasts.

**FIGURE 4:**
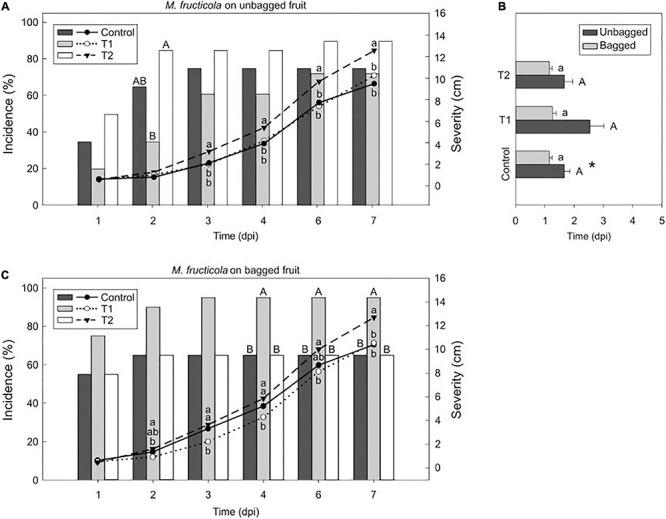
Light effect on the *M. fructicola*-nectarine interaction. Incidence (% of brown rot, bars) and severity (lesion diameter length in cm of rotted fruit, lines) of *M. fructicola* in unbagged **(A)** and bagged **(C)** “Fantasia” nectarines along the infection time course (dpi, days post-inoculation) incubated for 7 days under treatments 1 and 2 and control condition (constant darkness). Bars represent the mean of incidence on fruits (*n* = 20). Lines represent the mean of diameter length of rotted fruit. Different uppercase and lowercase letters indicate significant differences (*P* ≤ 0.05) of incidence and severity, respectively, among incubation conditions according to orthogonal contrasts at each time point. No letters indicate no significant differences. The incubation period (dpi) of *M. fructicola* in bagged and unbagged “Fantasia” nectarines **(B)** after 7 days of incubation under treatments 1 and 2 and control condition. Bars represent the mean of fruits with symptoms (*n* = 2 to 20) and error bars represent the standard error of the means. Different uppercase and lowercase letters indicate significant differences (*P* ≤ 0.05) among incubation conditions in unbagged and bagged fruit, respectively, according to orthogonal contrasts. Symbol (*) indicates significant differences between bagging conditions under control condition according to Student’s *T*-test (*P* ≤ 0.05).

To further investigate the light effect in brown rot progress at postharvest, we assessed some aggressiveness features after incubating the inoculated fruit under the lighting treatments. Results demonstrated that the effect of light on the host-pathogen interaction was cultivar-dependent. While we observed significant differences in incidence and severity of early-mid cultivars such as “Fantasia” (Figures 3A,C, 4A,C), we detected almost no differences in late cultivars such as “Nectatinto” and “Albared” (data not shown). The disease behavior on the later cultivars was similar among all lighting treatments. In addition, the incubation period was slightly higher in the early cultivars than in the late ones (data not shown).

Afterward, we selected the “Fantasia” cultivar for further analysis. *Monilinia laxa* incidence on unbagged nectarines maintained under control condition (constant darkness) was significantly higher (84 %) than that under treatment 1 and 2 (42 % and 35 %, respectively) (Figure 3A). The lesion diameter revealed the same tendency as incidence, although with no significant differences along time (Figure 3A). In the same line, the incubation period was significantly higher in inoculated unbagged fruit exposed to the treatment 2 than treatment 1 and control condition (1.5- and 1.8-fold, respectively) (Figure 3B). Regarding nectarines that were bagged during preharvest, there was no difference in neither incidence (ranging from 40 to 65 %) (Figure 3C) nor incubation period (between 2 and 3 days) among treatments (Figure 3B). However, the severity of *M. laxa*-inoculated fruit subjected to both control condition and treatment 1 was significantly higher than that under treatment 2 at 3 and 4 dpi (Figure 3C), although such differences subsided along the infection time course.

In ‘Fantasia’ unbagged nectarines inoculated with *M. fructicola*, the incidence at 2 dpi under treatment 2 (85 %) was significantly higher than those incidences under control condition and treatment 1 (65 % and 35 %, respectively). Treatment 2 also significantly increased severity (up to 12.6 cm) in unbagged fruit along time compared to that under control condition and treatment 1 (9.5 and 10.2 cm, respectively) (Figure 4A). However, the incubation period was similar among all treatments (Figure 4B). In bagged fruit, treatment 1 rose disease incidence (95 %) and was significant from 4 dpi onward, compared to the other treatments tested (65 % both). Contrary, treatment 2 significantly increased severity (12.7 cm) compared to that under control condition and treatment 1 (10.4 and 10.5 cm, respectively) (Figure 4C). No differences were observed among treatments when analyzing the incubation period of bagged or unbagged fruit (Figure 4B). Interestingly, the *M. fructicola*-incubation period of bagged fruit incubated under control condition was significantly lower (1.4- fold) than unbagged fruit at the same condition (Figure 4B). Overall, light seemed to negatively affect the disease incidence and severity of *M. laxa* whereas it caused the opposite effect for *M. fructicola*.

### Ethylene Production in *M. laxa*-Fruit Interaction Is Bag and Lighting Treatment-Dependent

**FIGURE 5:**
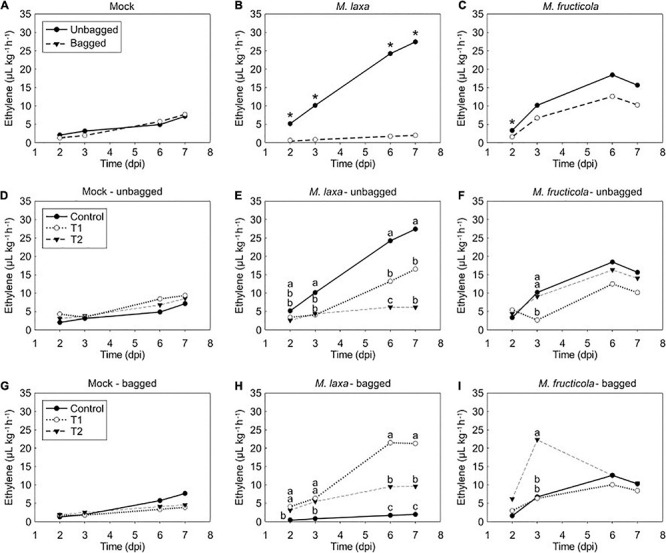
Ethylene production of mock fruit, *M. laxa*- and *M. fructicola*-fruit interaction among bag and lighting treatments through time in “Fantasia” nectarines. Ethylene measurements of mock **(A)**, *M. laxa*-fruit **(B)** and *M. fructicola*-fruit **(C)** along the infection time course (dpi, days post-inoculation) under control condition (constant darkness). Symbols (*) indicate significant differences between bagging conditions at each time point according to Student’s *T*-test (*P* ≤ 0.05). Ethylene measurements in mock fruit **(D,G)**, *M. laxa*-fruit **(E,H)** and *M. fructicola*-fruit **(F,I)** in unbagged **(D–F)** and bagged conditions **(G–I)** along the infection time course (dpi) under each lighting treatment. Different letters indicate significant differences (*P* ≤ 0.05) among light conditions according to orthogonal contrasts at each time point. No letters indicate no significant differences. In all graphics, values represent the mean of ethylene measurements of each replicate (*n* = 4).

In addition, to assess the development of the disease in *Monilinia* spp.-inoculated nectarines, we also evaluated the ethylene production of the pathosystem under the different experimental treatments (Figure 5). Firstly, we determined the bagging effect on ethylene production in *Monilinia*-inoculated fruit incubated under control condition (constant darkness) (Figures 5A–C). Results denoted that while unbagged and bagged mock-inoculated fruit produced similar ethylene levels (Figure 5A), on *M. laxa* inoculated nectarines, the levels of ethylene produced by the unbagged fruit were significantly higher than those in the bagged fruit (Figure 5B). Contrary to *M. laxa, M. fructicola* induced a peak of ethylene at 6 dpi in both unbagged and bagged fruit and results only showed significant differences between bagging conditions at 2 dpi (Figure 5C).

We further evaluated the light effect on both unbagged and bagged fruit inoculated with each species. Results demonstrated no significant differences in the ethylene production of mock-inoculated fruit among treatments regardless of the bagging condition in which come from Figures 5D,G. Ethylene levels of unbagged fruit inoculated with *M. laxa* were significantly higher at 7 dpi when incubated under control condition than when exposed to treatments 1 and 2 (4.5 and 2.7-fold, respectively) (Figure 5E). In contrast, bagged fruit inoculated with *M. laxa* and incubated under control condition displayed an opposite ethylene pattern (Figure 5H). Under lighting treatments, fruit inoculated with *M. laxa* slowly increased ethylene production of the pathosystem along time and was significantly higher than under control condition, resulting in a 10.9- and 4.9-fold increase under treatment 1 and 2, respectively. Regarding unbagged fruit inoculated with *M. fructicola*, all incubation treatments showed similar ethylene patterns, which peaked at 6 dpi. Only at 3 dpi, fruit incubated under treatment 1 significantly produced lower ethylene levels than the other treatments (Figure 5F). Bagged fruit inoculated with *M. fructicola* revealed a similar pattern to unbagged fruit. In that case, only ethylene levels of the *M. fructicola*-fruit interaction exposed to treatment 2 significantly peaked at 3 dpi (Figure 5I).

When comparing the ethylene emission pattern among mock-inoculated fruit and *Monilinia* spp. inoculated fruit on unbagged nectarines (Supplementary Figure 3), results clearly demonstrated that the ethylene pattern emitted by both *Monilinia* spp.-inoculated fruit incubated under control treatment was significantly higher than the one produced by mock fruit. The ethylene production of *M. laxa*-fruit interaction increased progressively along time, producing a similar pattern to mock fruit, although to a different extent, depending on the incubation treatment. In fact, *M. laxa*-inoculated fruit maintained under control treatment produced significantly higher levels (3.8-fold) than those of the mock fruit (Supplementary Figure 3A), while a slight difference of 1.8-fold between mock and *M. laxa*-inoculated fruit was observed under the treatment 1 at 7 dpi (not statistically different) (Supplementary Figure 3B). Conversely, the presence of *M. fructicola* stimulated an ethylene peak at 6 dpi that was 3.8, 1.5, and 1.6-fold higher under control treatment, treatment 1 and 2, respectively, if compared to mock-inoculated fruit (Supplementary Figure 3). Overall, both *Monilinia* spp. induced the ethylene levels of the pathosystem but in a lighting treatment-dependent manner.

## Discussion

Light is essential both in the preharvest period (i.e., solar radiation) and postharvest chain (i.e., artificial lighting) of fruit. The combination of light quality, intensity, and photoperiod constitute a source of information for the fruit but also to pathogens, and in turn, can influence the onset of symptoms of the development of the disease on the fruit surface. Scarce information regarding *in vitro* development of *Monilinia* spp. or brown rot infection on stone fruit under the effect of light is available. Some studies have been conducted with discrete sections of the spectrum such as long-wave UV (De Cal and Melgarejo, 1999), in other *Monilinia* spp. such as *M. fructigena* (Marquenie et al., 2003) and the effect of visible white light in *M. laxa* isolates (Rodríguez-Pires et al., 2020, 2021). However, a study aiming to decipher the effect of lighting treatments on the two main *Monilinia* spp. of stone fruit has never been conducted. Accordingly, we characterized for the first time, the effect of different lighting treatments on both the *in vitro* fungal development of *M. fructicola* and *M. laxa*, and during the interaction of *Monilinia* spp. – nectarine fruit, using similar artificial lighting treatment previously applied to *M. laxa*-stone fruit studies (Rodríguez-Pires et al., 2020).

### Altered Conidia Morphology Impairs the Conidial Viability in a *Monilinia* spp.-Dependent Manner

*Monilinia laxa* demonstrated a broader photomorphogenesis response to light than *M. fructicola* under *in vitro* conditions. In this study, cultures grown on either PDA or PDA-T media and incubated under control condition (constant darkness) were similar to other *M. laxa* or *M. fructicola* isolates grown on similar conditions (Tran et al., 2020; Rodríguez-Pires et al., 2021). After exposure to both lighting treatments, but especially under treatment 2, *M. laxa* mycelia turned mainly hazel whereas the colony color of *M. fructicola* was not altered at any condition (Figures 1A,B). To regulate fungal biology, fungi sense light through photoreceptors and use it as an input of information (Tisch and Schmoll, 2010). One of the most common and long-term effects of light responses is the induction of pigment expression, such as carotenoid biosynthesis in many microorganisms (Fuller et al., 2015; Corrochano, 2019), and, in fact, the carotenoid production in the closely related organism *B. cinerea* has been suggested (Schumacher et al., 2014). In turn, carotenoids are highly implicated in protecting cells from reactive oxygen species (ROS) (Avalos and Limón, 2015). Light also induces the biosynthesis of other pigments such as melanin and mycosporines in several fungi (Fuller et al., 2015). The role of melanin in *M. fructicola* has been described on not only the protection against environmental stresses such as desiccation, UV irradiation, and temperature (Rehnstrom and Free, 1996), but also on the conidia turgor adjustment and full virulence to infect stone fruit (Yu et al., 2020). Visible light can cause oxidative stress in *B. cinerea* cells, which could be, in part, due to an alteration in the homeostasis of cellular ROS levels (Canessa et al., 2013). In fact, our results revealed how both treatments altered the morphology of conidia after 7 days of incubation under each light condition (Figures 1A,B) if compared to typically limoniform (or also cylindrical in the case of *M. laxa*) conidia shapes (Yin et al., 2015) observed under control condition. Therefore, these findings suggest that under these lighting treatments, conidia were submitted to stress that ultimately affected cell turgor. However, the impaired morphology could also rely on the result of the phototropism generated in response to light, which has been described in conidia, apothecia and conidial germ tubes of *B. cinerea* (Jarvis, 1972). Regarding conidial viability, studies on how light alters the ability to form new colonies of *Monilinia* spp. are nonexistence. We demonstrated that *M. laxa*, but not *M. fructicola*, increased its conidial viability under treatment 1 but reduced it under treatment 2 in relation to control condition (Figures 1C,D). In fact, Lafuente et al. (2018) already demonstrated that continuous blue light and complete darkness increased *Penicillium digitatum* cell viability *in vitro* compared to non-continuous light. These results are in line with what we observed for *M. laxa*, since the spectrum of lights used in this study do emit small wavelengths around blue. Alternatively, the altered conidia morphology could explain the reduction of *M. laxa* cell viability under treatment 2. Thus, the relation between turgor and the ability to form new colonies is a point of interest. Although some studies point out the role of light in controlling the conidial germination (Corrochano, 2019; Yu and Fischer, 2019), herein we did not observe an effect either on *M. laxa* or *M. fructicola* (Figures 1E,F).

### *Monilinia laxa* Coped With Light Stress and Its *in vitro* Development Was Favored

Light altered the *in vitro* fungal expansion, especially in *M. laxa*. Under standard conditions (growing on PDA medium at 22–25°C and darkness), *M. fructicola* grows faster and produces more conidia than *M. laxa* (Villarino et al., 2016; Tran et al., 2020), like observed in the present study (Figure 1). However, lighting treatments made *M. laxa* to grow and produce more conidia (compared to control condition) than *M. fructicola* on PDA-T medium. Another reported light effect, widely described in *B. cinerea* (Schumacher, 2017), is that light can regulate biological responses, such as vegetative mycelial growth and the transition from sexual to asexual reproduction (conidiation) (Corrochano, 2019). In fact, the endogenous circadian clock also controls conidiation (Hevia et al., 2015). In this line, Canessa et al. (2013) reported that a photoperiod of cool white light and control condition reduced the growth rate and increased conidiation of a strain of *B. cinerea*. Our results suggest that *M. laxa* and *M. fructicola* behaved similarly to *B. cinerea* in terms of conidiation and growth rate, respectively. *Botrytis cinerea* perceives and reacts to the entire visible spectrum and beyond, and several fungal biological responses have been described for each monochromatic section of the spectrum (Schumacher, 2017; Veloso and van Kan, 2018). Green light (around 540 nm) represses mycelial growth (Zhu et al., 2013), whereas blue (around 450 nm) and red (around 650 nm) light restrain conidiation (Tan, 1975). Both treatment 1 and 2 tested herein emit three wavelength peaks around 440, 550, and 630 nm. Remarkably, the orange/red wavelength of treatment 1 is higher than the treatment 2 one. Hence, although *M. laxa* is able to sense and express green light photoreceptors (Rodríguez-Pires et al., 2021), its growth was increased rather than repressed. Zhu et al. (2013) found that under green light, *B. cinerea* cells showed deformed mitochondria and enlarged central vacuoles, probably as a result of the vacuoles’ action to eliminate cell structures damaged due to the light stress (Shoji et al., 2010), and in consequence, the growth rates of *B. cinerea* were retarded. However, under such light stress, *M. laxa* could be coping with it through autophagy of damaged organelles structures to support mycelial growth, as has been demonstrated when nutrient availability is limited (Shoji et al., 2010). A contrary effect was observed for *M. fructicola* which suggests the different ability of both species to sense and respond to light. The mechanisms underlying such differences are encouraged. An example pathway of interest related to light is the light-responsive transcription factor (LTF1), which controls development but also is required for maintenance of the redox homeostasis in mitochondria and full virulence in *B. cinerea* (Schumacher et al., 2014). Overall results showed that growth rate was in line with conidial viability and the reviewed results evidence the different ability of both *Monilinia* spp. to cope with lighting treatments.

Blue and red light have been described to repress conidiation in *B. cinerea* (Tan, 1975). Thus, although *M. laxa* is able to sense and express blue and red-light photoreceptors (Rodríguez-Pires et al., 2021), it increased its conidiation, whereas the conidia production of *M. fructicola* seemed to be affected by these sections of the spectrum (Figures 1C,D). Recent studies have shown that red light drastically increases conidiation of *M. laxa* compared to control condition (constant darkness) while does not affect or alter *M. fructicola* conidiation when compared to control condition (Verde-Yáñez et al., unpublished). Conidiation is regulated by light-responsive transcription factors, such as FL (*fluffy*) for undifferentiated mycelia, and it is induced by blue light through the blue-light photoreceptor WHITE COLLAR COMPLEX in the fungal model *Neurospora crassa* (Olmedo et al., 2010). However, our results revealed a fluffy phenotype of *M. laxa* when growing on PDA-T medium and incubated under control condition. Hence, other transcription factors should be responsible for the increased conidiation in *M. laxa* and in-depth studies should be conducted. Blue light has also been shown to act as an antimicrobial agent (Kahramanoğlu et al., 2020), which could, in part, explain the reduced conidiation observed in *M. fructicola*, highlighting again the different ability of both species to respond to light.

### The Light-Induced Impaired Fungal Development Ultimately Alters Their Capacity to Infect Fruit

Light also affects the ability of pathogens to infect and rot fruit, such as described in several pathosystems (Islam et al., 1998; Lafuente et al., 2018). Among the aspects of fungal behavior and development that light can govern, light can regulate secondary metabolism, also related to the balance between sexual development toward conidia (Tisch and Schmoll, 2010; Schumacher, 2017). Our results demonstrated that after incubating *M. laxa* and *M. fructicola* under each lighting treatment for 7 days prior to fruit inoculation, both treatments reduced the ability of *M. laxa* to infect fruit, whereas only treatment 1 seemed to increase the virulence of *M. fructicola* (Figure 2). The colored phenotype and/or the altered conidia morphology observed in *M. laxa* grown on PDA-T medium maintained under lighting treatments could in part, explain its reduced capacity to infect. Similar results and hypotheses have been described for the *P. digitatum*-orange pathosystem. In line with spore viability, continuous blue light (450 nm) and complete darkness exposition of *P. digitatum* cultures lead to increased capability to infect oranges if compared to cultures submitted to non-continuous light (Lafuente et al., 2018). The authors suggested that the anomalous morphology of spores was more responsible for the lower capacity to infect fruit rather than the other parameters evaluated (metabolic activity and ethylene production). Alternatively, mutants of *B. cinerea* producing conidia in either light or darkness are associated with reduced virulence in primary leaves of French bean (Schumacher et al., 2012). However, how these altered features ultimately impair viability and capacity to infect fruit needs further investigation. Interestingly, fruit inoculated with *M. laxa*, previously incubated under treatment 1, showed conidia on fruit surface only after 7 dpi, slightly later than under control conditions (constant darkness) (6 dpi) and no conidiation was observed under treatment 2 (Figure 2B). In the line with what observed in *P. digitatum*, opposite incubation conditions (continuous light *vs.* complete darkness) can induce similar fungal phenotypes and responses, such as those observed herein regarding *M. laxa*. Contrary to *M. laxa*, in *M. fructicola*, the effect of light was mainly observed at the beginning of the infection course, showing the highest diameter length, accelerating the appearance of the onset of brown rot symptoms, and inducing more conidia on the fruit surface (Figures 2C,D and Supplementary Figure 1). Overall, results suggest that altered conidia morphology and reduced *in vitro* conidiation could positively impair its virulence on the fruit surface. Studies regarding the effect of light in photoreceptors related to conidiation (blue and red) and their signaling cascade would be interesting to be evaluated prior to and after fruit infection.

### The Development of the Disease Relays on the Pathogen’s Light Effect Rather Than on the Fruit Itself

Plants are continuously exposed to a variety of abiotic stresses, which could drive to a modulation of the plant phenotype. Light is one of the major and influential inputs for their physiology and is perceived through plant photoreceptors (Folta and Carvalho, 2015). For that reason, in response to light, the mechanisms to face biotic stresses can also be altered. When *Monilinia* spp.-inoculated unbagged fruit were incubated under each treatment (Figures 3A,B, 4A,B), results revealed a comparable fungal development than the one observed when the pathogens were previously incubated under each lighting treatment prior to fruit inoculation. Both lighting treatments reduced *M. laxa* incidence, whereas control condition (constant darkness) reduced *M. fructicola* in unbagged fruit, elucidating that the fruit responses were *Monilinia* spp. dependent rather than dependent on light conditions. In *Arabidopsis thaliana* plants inoculated with *B. cinerea*, constant light and a photoperiod of light/dark considerably reduced the lesion areas compared to constant darkness (Canessa et al., 2013), in concordance with what we observed in *M. laxa*. Similar to that described in fungi, plant photoreceptors also perceive narrow-bandwidth wavelengths, which in turn activate specific internal responses (Folta and Carvalho, 2015). For instance, Zhu et al. (2013) demonstrated that white and green light decreased lesion diameter in *B. cinerea*-inoculated grapes and only green light reduced diameter in *B. cinerea*-inoculated tomatoes. Herein, we demonstrated that light had a major effect on *Monilinia* spp. rather than on fruit integrity, suggesting that pathogens are differentially modulating fruit responses.

### Preharvest Fruit Conditions Influence the Disease Plant Response

Preharvest conditions are also crucial for fruit integrity and in turn, in its capacity to face any stress. Fruit bagging is an emerging agricultural practice mainly down to reduce the amount of fungicide on fruit surface. Bagging the fruit alters the solar radiation that irradiates fruit, and hence, influencing internal quality parameters (Sharma et al., 2014) skin color (Zhou et al., 2019) and marketable yield at harvest (Allran, 2017). Therefore, fruit bagging may result in changed defense response against pathogens. Herein, while bagged “Fantasia” cultivar was less susceptible to brown rot, bagged fruit of the other cultivars were more susceptible to both *Monilinia* spp. under control condition (Supplementary Table 1). Hence, findings point out that different solar radiation received by the unbagged and bagged fruit can differentially affect the fruit defense mechanisms in front of brown rot in a cultivar-dependent manner. Several studies conducted to test the bagging effect have also shown contradictory results when comparing cultivars, and fruit- and cultivar-specific responses have been suggested as one of the main causes (Sharma et al., 2014). In fact, fruit have different intrinsic characteristics depending on the stone fruit cultivar that leads to a different brown rot susceptibility (Baró-Montel et al., 2019a; Obi et al., 2019). Out of the responses of the host to counteract the pathogen’s intrusion, fruit activates stress responses through activating the antioxidant metabolism such as glutathione and redox-related amino acids (Balsells-Llauradó et al., 2020). Hence, analyzing intrinsic properties differing among cultivars such as quality parameters and fruit antioxidant metabolism, could ultimately be correlated with brown rot development, and thus, could shed light on the incidence differences among cultivars. In addition to that, fruit bagging can also affect to microclimate around the fruit, increasing temperature and humidity and in turn, affecting to transpiration, respiration and cuticle in peel cells (Ali et al., 2021).

The development of *Monilinia* spp. in bagged fruit, incubated under each incubation treatment (Figures 3C, 4C), was slightly different from the one observed in unbagged fruit. Hence, preventing the fruit from solar radiation may have caused not only an impact on the fruit’s intrinsic characteristics but also on the response to face the pathogens. Therefore, results highlight not only the importance of the light effect in preharvest (solar radiation), but also its effect in postharvest (artificial lighting). Solar light comprises a broad range of electromagnetic waves. The red light fraction of the spectra is of interest since not only was suggested to alter the behavior of *Monilinia* spp. (Section “Altered conidia morphology impair the conidial viability in a *Monilinia* spp.-dependent manner” and “*Monilinia laxa* coped with light stress and its *in vitro* development was favored”), but it can also have a positive effect on fruits in front of *M. laxa*, but not in front of *M. fructicola*. For instance, the previous incubation of strawberry leaves under red light significantly increased its resistance to *B. cinerea* (Meng et al., 2019). Further from the visible light, UV-C irradiation can induce resistance in several fruit and vegetables (reviewed in Romanazzi et al., 2016). Light quality can strongly modulate phenolic compounds, flavonoids, carotenoids, and anthocyanins (reviewed in Ilić and Fallik, 2017), being chlorophyll and carotenoids directly activated by photons. In particular, the activation of phenylpropanoids biosynthesis is enhanced by light in *Xanthomonas oryza*-treated rice leaves (Guo et al., 1993) and by the combination of red and blue light in lettuce (Heo et al., 2012). In addition, the expression of the zeaxanthin epoxidase, a flavoprotein from the carotenoid biosynthesis, that is active under light (Latowski et al., 2000), is upregulated in inoculated-fruit with *M. laxa* compared to healthy fruit along time (Balsells-Llauradó et al., 2020). Accordingly, future studies aiming to unravel the different fruit properties such as secondary metabolites in response to light would contribute to a better understanding of the fruit’s capability to face the pathogens.

### Ethylene Production in the Host-Pathogen Interaction Is Mainly Influenced by *Monilinia* spp. Rather Than the Bag and Light Effect

Ethylene has been implicated in modulating the plant response not only to abiotic stresses but also to necrotrophic pathogens (McDowell and Dangl, 2000; Müller and Munné-Bosch, 2015). Hence, ethylene modulations induced by fruit bagging, lighting treatments and *Monilinia* spp. were assessed on the nectarine-*Monilinia* spp. interaction. Several studies have described that light affects ethylene levels and other hormones (e.g., cytokinins) and suggest a crosstalk among light and both hormones (reviewed in Zdarska et al., 2015), influencing plant development. However, our results showed that the ethylene produced by mock-inoculated fruit was affected by neither the bag nor the lighting treatments analyzed (Figures 5A,D,G). Specifically, ethylene emission increased along time, following the production pattern of a climacteric fruit until ripening (Oetiker and Yang, 1995). In other crops, such as grapes, lighting treatment does neither induce ethylene compared to dark (Zhu et al., 2012). Only Gong et al. (2015) found that blue light can induce changes in ethylene production to accelerate postharvest ripening in peaches, although the lighting treatments tested herein only emit a short intensity of blue light wavelength. Hence, ‘Fantasia’ cultivar was not affected by these abiotic conditions in terms of ethylene production.

Some fungi can also produce ethylene although its function in fungal development or as a virulence factor is inconclusive (Chague, 2010). Recently, white and blue lights have been shown to significantly increase the ethylene production rate of several fungi (such as *B. cinerea*) under *in vitro* conditions compared to dark, and that even *B.* cinerea could be the ethylene producer in an interaction with *A. thaliana* seedlings (Guo et al., 2020). However, the ethylene production by *Monilinia* spp. has not been deciphered to date. Herein, overall changes in the ethylene pattern of the pathogen-fruit pathosystem were due to the interaction with the pathogen and to bagging and lighting treatments. Among all the host responses that plant ethylene mediates (McDowell and Dangl, 2000), this hormone is also implicated in ripening and senescence processes, which can be conducive to disease susceptibility (Liu et al., 2015; Pandey et al., 2016). In fact, a different ethylene pattern was observed for both pathogens in interaction with fruit (Figure 5 and Supplementary Figure 3), pointing out to either a different response of the host to cope with the two *Monilinia* spp. or a different *Monilinia* species-dependent modulation to avoid the ethylene-mediated defense response. Other studies also reported a different modulation of ethylene production by both *Monilinia* spp.-fruit interaction in artificially inoculated peaches (Baró-Montel et al., 2019b) and peach petals (Vall-llaura et al., 2020). Although several hypotheses have been suggested, its role in promoting defense or susceptibility is still controversial (van Loon et al., 2006). In addition, results highlighted that the incubation under lighting treatments and the presence of the bag did alter the ethylene production, especially in *M. laxa*-inoculated fruit. These results could in turn explain the altered fruit’s capability to respond to these species (Figures 5B,E,H and Supplementary Figures 3A,B). Accordingly, unbagged fruit incubated under control condition demonstrated an increased *M. laxa* incidence and a lower incubation period (Figure 3), revealing that this species took advantage of the increased ethylene production. However, in *M. laxa*-inoculated bagged fruit both lighting treatments significantly induced ethylene production, such as the ethylene-induced in the *B. cinerea*-grapes pathosystem (Zhu et al., 2012). In addition to the plant ethylene role in biotic interactions, Xiang et al. (2020) suggested that the main downstream regulators of phytochromes (the phytochrome-interacting factors, PIFs) acted upstream of the ethylene response factor 1 (ERF1) to negatively regulate the resistance to *B. cinerea* in *A. thaliana*. With that, those authors suggested that the PIF-mediated defense against the pathogen is closely related to the jasmonate/ethylene signaling pathway. Thus, molecular studies related to the signaling downstream phytochromes need further investigation to understand the dual ethylene responses occurring during the nectarine-*Monilinia* spp. interaction under light conditions.

## Concluding Remarks

To avoid or delay the appearance of brown rot symptoms and conidiation on the fruit surface and hence, reduce economic losses driven from contamination through conidia spreading along the postharvest chain, environmental light conditions should be considered. Our study highlights the different behavior of *M. laxa* and *M. fructicola* in both *in vitro* and *in vivo* development and further studies aiming to investigate the differences that underlie the impaired photomorphogenesis due to lighting treatments, such genes related to conidiation, of both species is encouraged. White lighting treatment has not only impaired the fungal development but also the host response to the pathogen attack. Light received for the fruit during preharvest modifies its intrinsic properties that ultimately would influence its capability to prevent or overcome the infection caused by *Monilinia* spp. During postharvest, light incidence also affected the nectarine-*Monilinia* spp. interaction since fungal development was altered in a species-dependent manner. Thus, deciphering the light-dependent modulation of the fruit properties that will give rise to improved defense response, but also the light-effect that triggers fungal development, will allow contributing to the development of new strategies to control brown rot at both preharvest and postharvest.

## Data Availability Statement

The original contributions presented in the study are included in the article/Supplementary Material, further inquiries can be directed to the corresponding author.

## Author Contributions

JU, RT, and NV conceived and designed the experiments. MB-L, NT, and CC carried out fruit inoculations, *in vitro* studies, pathological studies, and ethylene measurements. MB-L, NV, and RT wrote the article. CC, NT, and JU contributed to improving the final version of the manuscript. All authors contributed to the article and approved the submitted version.

## Conflict of Interest

The authors declare that the research was conducted in the absence of any commercial or financial relationships that could be construed as a potential conflict of interest.

## Publisher’s Note

All claims expressed in this article are solely those of the authors and do not necessarily represent those of their affiliated organizations, or those of the publisher, the editors and the reviewers. Any product that may be evaluated in this article, or claim that may be made by its manufacturer, is not guaranteed or endorsed by the publisher.

## Supplementary Material

Supplementary Figure 1 Conidiation of *M. fructicola* on “Fantasia” cultivar surface. The concentration of conidia is represented relative to control condition (dark). Different letters indicate statistically differences among treatments according to orthogonal contrasts (*P* < 0.05).

Supplementary Figure 2 Light effect on the capacity of *Monilinia* spp. to infect fruit in “Venus” cultivar. Incidence (% of brown rot, bars) and severity (lesion diameter length in cm of rotted fruit, lines) of *M. laxa* **(A)** and *M. fructicola* **(C)** in “Venus” nectarines along the infection time course (dpi, days post inoculation) after growing the fungi during 7 days under control and treatments 1 and 2. Bars represent the mean of incidence on fruit (*n* = 20). Lines represent the mean of diameter length of rotted fruit. Different uppercase and lowercase letters indicate significant differences (*P* ≤ 0.05) of incidence and severity, respectively, among treatments according to orthogonal contrasts at each time point. No letters indicate no significant differences. Incubation and latency periods (days) of *M. laxa* **(B)** and *M. fructicola* **(D)** in “Venus” nectarines after growing the fungi during 7 days under control and treatments 1 and 2. Bars represent the mean of fruits with symptoms (*n* = 1 to 20) and error bars represent the standard error of the means. Different lowercase and uppercase letters indicate significant differences (*P* ≤ 0.05) of incubation and latency periods, respectively, among treatments according to orthogonal contrasts. Conidiation of *M. fructicola* on fruit surface **(E)**. The concentration of conidia is represented relative to control (dark). Different letters indicate statistically differences among treatments according to orthogonal contrasts (*P* < 0.05).

Supplementary Figure 3 Ethylene production of mock-inoculated fruit, *M. laxa* and *M. fructicola*-inoculated fruit on unbagged nectarines. Ethylene measurements of mock, *M. laxa*, *M. fructicola*-inoculated fruit incubated during 7 days under control condition **(A)** and treatments 1 **(B)** and 2 **(C)**. Different letters indicate significant differences among inoculums at each time point according to Tukey’s HSD test (*P* ≤ 0.05). No letters indicate no significant differences. In all graphics, values represent the mean of ethylene measurements of each replicate (*n* = 4).

Supplementary Table 1 *Monilinia* spp. incidence (%) on unbagged and bagged fruit from different cultivars after 7 days of incubation under control condition (dark). No significant differences between bagging conditions were found according to generalized linear model (GLM).
