# Supplementary material for: Light Intensity Alters the Behavior of Monilinia spp. in vitro and the Disease Development on Stone Fruit-Pathogen Interaction
Source: Front Plant Sci. 2021 Sep 8;12:666985. doi: 10.3389/fpls.2021.666985 (PMC8455894; doi:10.3389/fpls.2021.666985)
Supplement: Supplementary Figure 1 — Conidiation of M. fructicola on “Fantasia” cultivar surface. The concentration of conidia is represented relative to control condition (dark). Different letters indicate statistically differences among treatments according to orthogonal contrasts (P < 0.05). [file Data_Sheet_1.zip › Supplementary Figure S1.DOCX]

Supplementary Material

**
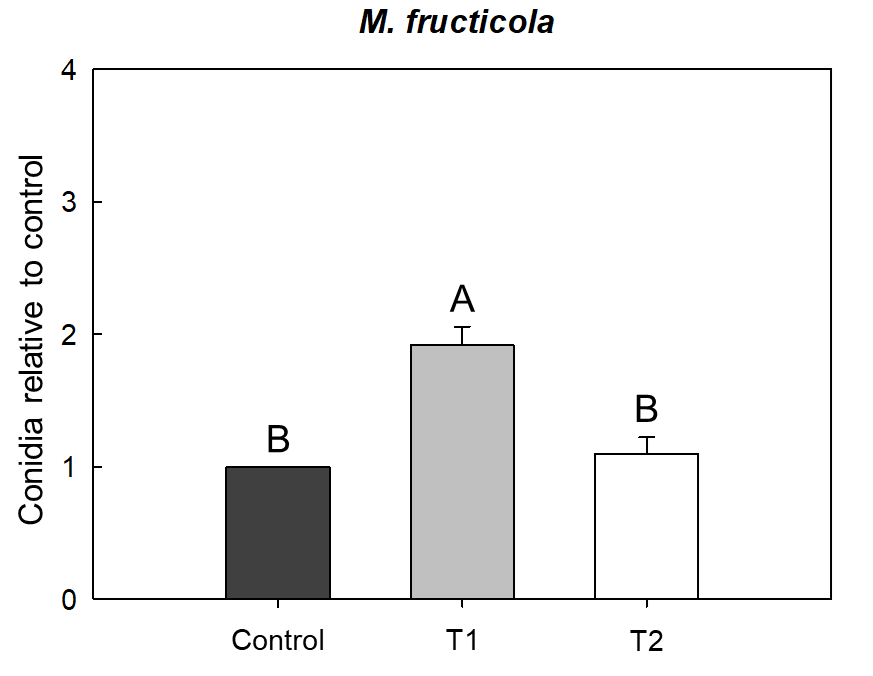
**

**Supplementary Figure S1. Conidiation of *M. fructicola* on ‘Fantasia’ cultivar surface*.*** The concentration of conidia is represented relative to control condition (dark). Different letters indicate statistically differences among treatments according to orthogonal contrasts (*P* < 0.05).
