# Supplementary material for: Light Intensity Alters the Behavior of Monilinia spp. in vitro and the Disease Development on Stone Fruit-Pathogen Interaction
Source: Front Plant Sci. 2021 Sep 8;12:666985. doi: 10.3389/fpls.2021.666985 (PMC8455894; doi:10.3389/fpls.2021.666985)
Supplement: Supplementary Figure 1 — Conidiation of M. fructicola on “Fantasia” cultivar surface. The concentration of conidia is represented relative to control condition (dark). Different letters indicate statistically differences among treatments according to orthogonal contrasts (P < 0.05). [file Data_Sheet_1.zip › Supplementary Figure S2.DOCX]

Supplementary Material


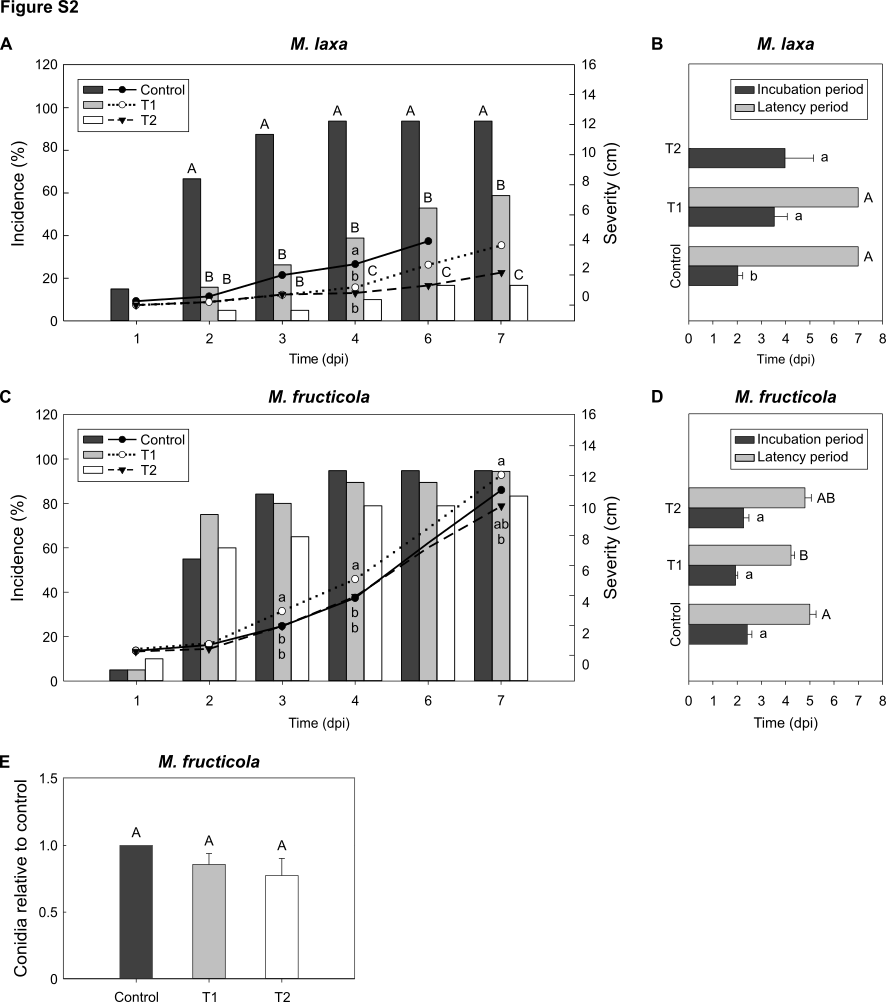


**Supplementary Figure S2**. **Light effect on the capacity of *Monilinia* spp. to infect fruit in ‘Venus’ cultivar**. Incidence (% of brown rot, bars) and severity (lesion diameter length in cm of rotted fruit, lines) of *M. laxa* **(A)** and *M. fructicola* **(C)** in ‘Venus’ nectarines along the infection time course (dpi, days post inoculation) after growing the fungi during 7 days under control and treatments 1 and 2. Bars represent the mean of incidence on fruit (n = 20). Lines represent the mean of diameter length of rotted fruit. Different uppercase and lowercase letters indicate significant differences (*P* ≤ 0.05) of incidence and severity, respectively, among treatments according to orthogonal contrasts at each time point. No letters indicate no significant differences. Incubation and latency periods (days) of *M. laxa* **(B)** and *M. fructicola* **(D)** in ‘Venus’ nectarines after growing the fungi during 7 days under control and treatments 1 and 2. Bars represent the mean of fruits with symptoms (n = 1 to 20) and error bars represent the standard error of the means. Different lowercase and uppercase letters indicate significant differences (*P* ≤ 0.05) of incubation and latency periods, respectively, among treatments according to orthogonal contrasts. Conidiation of *M. fructicola* on fruit surface (**E**)*.* The concentration of conidia is represented relative to control condition (dark). Different letters indicate statistically differences among treatments according to orthogonal contrasts (*P* < 0.05).
