# Supplementary material for: Light Intensity Alters the Behavior of Monilinia spp. in vitro and the Disease Development on Stone Fruit-Pathogen Interaction
Source: Front Plant Sci. 2021 Sep 8;12:666985. doi: 10.3389/fpls.2021.666985 (PMC8455894; doi:10.3389/fpls.2021.666985)
Supplement: Supplementary Figure 1 — Conidiation of M. fructicola on “Fantasia” cultivar surface. The concentration of conidia is represented relative to control condition (dark). Different letters indicate statistically differences among treatments according to orthogonal contrasts (P < 0.05). [file Data_Sheet_1.zip › Supplementary Figure S3.DOCX]

Supplementary Material


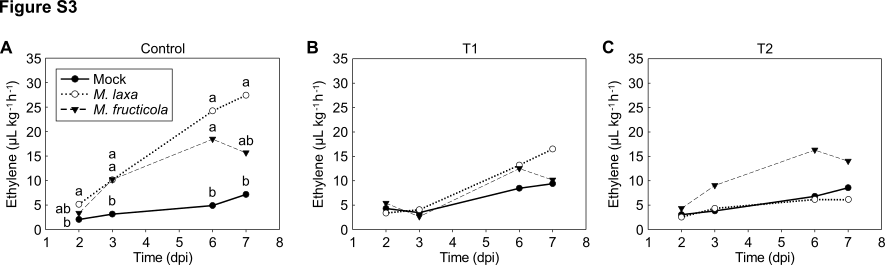


**Supplementary Figure S3**. **Ethylene production of mock-inoculated fruit, *M. laxa* and *M. fructicola*-inoculated fruit on unbagged nectarines.** Ethylene measurements of mock, *M. laxa*, *M. fructicola-*inoculated fruit incubated during 7 days under control condition (**A**) and treatments 1 (**B**) and 2 (**C**). Different letters indicate significant differences among inoculums at each time point according to Tukey’s HSD test (*P* ≤ 0.05). No letters indicate no significant differences. In all graphics, values represent the mean of ethylene measurements of each replicate (n = 4).
