# Supplementary material for: Light Intensity Alters the Behavior of Monilinia spp. in vitro and the Disease Development on Stone Fruit-Pathogen Interaction
Source: Front Plant Sci. 2021 Sep 8;12:666985. doi: 10.3389/fpls.2021.666985 (PMC8455894; doi:10.3389/fpls.2021.666985)
Supplement: Supplementary Figure 1 — Conidiation of M. fructicola on “Fantasia” cultivar surface. The concentration of conidia is represented relative to control condition (dark). Different letters indicate statistically differences among treatments according to orthogonal contrasts (P < 0.05). [file Data_Sheet_1.zip › Supplementary Table S1.DOCX]

Supplementary Material

**Supplementary Table S1. *Monilinia* spp. incidence (%) on unbagged and bagged fruit from different cultivars after 7 days of incubation under control condition (dark).** No significant differences between bagging conditions were found according to generalized linear model (GLM).

| Cultivar | *Monilinia* spp. | Fruit condition | Incidence |
| --- | --- | --- | --- |
| ‘Fantasia’ | *M. laxa* | Unbagged | 84.2 % |
| ‘Fantasia’ | *M. laxa* | Bagged | 65.0 % |
| ‘Fantasia’ | *M. fructicola* | Unbagged | 75.0 % |
| ‘Fantasia’ | *M. fructicola* | Bagged | 65.0 % |
| ‘Venus’ | *M. laxa* | Unbagged | 15.0 % |
| ‘Venus’ | *M. laxa* | Bagged | 26.3 % |
| ‘Venus’ | *M. fructicola* | Unbagged | 82.4 % |
| ‘Venus’ | *M. fructicola* | Bagged | 89.5 % |
| ‘Nectatinto’ | *M. laxa* | Unbagged | 70.0 % |
| ‘Nectatinto’ | *M. laxa* | Bagged | 93.75 % |
| ‘Nectatinto’ | *M. fructicola* | Unbagged | 89.5 % |
| ‘Nectatinto’ | *M. fructicola* | Bagged | 94.4 % |
| ‘Albared’ | *M. laxa* | Unbagged | 75.0 % |
| ‘Albared’ | *M. laxa* | Bagged | 100.0 % |
| ‘Albared’ | *M. fructicola* | Unbagged | 100.0 % |
| ‘Albared’ | *M. fructicola* | Bagged | 100.0 % |
